# Detector-grade perovskite single-crystal wafers via stress-free gel-confined solution growth targeting high-resolution ionizing radiation detection

**DOI:** 10.1038/s41377-023-01129-y

**Published:** 2023-04-03

**Authors:** Yilong Song, Lixiang Wang, Yongqiang Shi, Weihui Bi, Jianwu Chen, Mingwei Hao, Anran Wang, Xueying Yang, Yuan Sun, Fan Yu, Liansheng Li, Yanjun Fang, Deren Yang, Qingfeng Dong

**Affiliations:** 1 State Key Laboratory of Supramolecular Structure and Materials, College of Chemistry, Jilin University, Changchun, 130012 China; 2 State Key Laboratory of Silicon Materials and School of Materials Science and Engineering, Zhejiang University, Hangzhou, 310027 China; 3 Beijing Institute of Control Engineering, Beijing, 100190 China; 4 Science and Technology on Space Intelligent Control Laboratory, Beijing, 100190 China; 5 Shanxi-Zheda Institute of Advanced Materials and Chemical Engineering, Taiyuan, 030024 China

**Keywords:** Imaging and sensing, X-rays

## Abstract

Solution-processed organic‒inorganic halide perovskite (OIHP) single crystals (SCs) have demonstrated great potential in ionizing radiation detection due to their outstanding charge transport properties and low-cost preparation. However, the energy resolution (ER) and stability of OIHP detectors still lag far behind those of melt-grown inorganic perovskite and commercial CdZnTe counterparts due to the absence of detector-grade high-quality OIHP SCs. Here, we reveal that the crystallinity and uniformity of OIHP SCs are drastically improved by relieving interfacial stress with a facial gel-confined solution growth strategy, thus enabling the direct preparation of large-area detector-grade SC wafers up to 4 cm with drastically suppressed electronic and ionic defects. The resultant radiation detectors show both a small dark current below 1 nA and excellent baseline stability of 4.0 × 10^−8 ^nA cm^−1^ s^−1^ V^−1^, which are rarely realized in OIHP detectors. Consequently, a record high ER of 4.9% at 59.5 keV is achieved under a standard ^241^Am gamma-ray source with an ultralow operating bias of 5 V, representing the best gamma-ray spectroscopy performance among all solution-processed semiconductor radiation detectors ever reported.

## Abstract (web summary)

The large-size detector-grade CH_3_NH_3_PbI_3_ single-crystal wafers were fabricated through stress-free gel-confined solution growth process, achieving an energy resolution of 4.9% under ^241^Am gamma-ray source with ultralow operating bias of 5 V.

## Graphical abstract

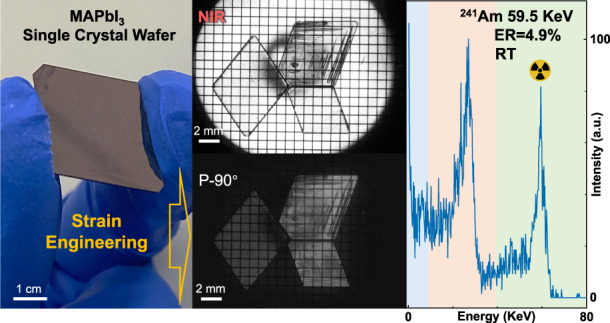

## Introduction

High-performance solid-state radiation detectors have been extensively exploited in a broad range of applications, such as medical diagnostic and therapeutic applications, industrial examination, scientific research and military defense^1–3^. Based on the device operational principle, they can be classified as indirect- and direct-type radiation detectors. Indirect-type detectors are composed of visible photodetectors covered with scintillators that convert high-energy photons into visible photons, yet the conversion efficiency remains low, which results in weak radioluminescence. In addition, due to the large ionization energy loss of the scintillation process, the energy resolution (ER) of indirect-type detectors is generally low. In contrast, direct-type detectors generate charge carriers directly under irradiation based on the photovoltaic and photoconductive effect of semiconductors and thus can potentially achieve both high charge conversion efficiency and ER.

Currently, commercial direct-type high-energy gamma-ray spectroscopy mainly relies on Cd_1–*x*_Zn_*x*_Te (CZT) single crystal (SC)-based detectors, which can be operated at room temperature and exhibit both high gamma-ray attenuation capability and excellent ER^4,5^. However, CZT detectors suffer from high material and manufacturing costs, as well as a high-temperature fabrication process that poses challenges to their direct integration with read-out circuitry. In addition, CZT detectors generally require special device designs to compensate for intrinsically unbalanced electron-hole transport properties, which complicates the device fabrication process. Moreover, a large working bias voltage of up to ~1000 V is necessary to achieve the full extraction of photon-generated charge carriers, which poses additional challenges for the power supply and dielectric protection. Therefore, it is highly desirable to explore new materials for the preparation of low-cost yet high-performance radiation detectors.

In the past few years, metal halide perovskites (MHPs) have stood out for their extraordinary potential for direct-type radiation detection due to their unique physical properties, including their large atomic number, large and balanced electron-hole mobility, long carrier recombination lifetime and exceptional defect tolerance properties^6–13^. Since the first demonstration of the gamma-ray response in melt-grown inorganic cesium lead bromide SCs in 2013^14^ as well as the gammavoltaic effect in solution-processed organic‒inorganic hybrid perovskite (OIHP) MAPbI_3_ SCs (MA stands for methylammonium) in 2015^6^, the MHP SCs have gained rapid development in the field of gamma-ray detection and have exhibited promising detection capability^3,15^. Kanatzidis’s group achieved a high ER of 3.8% for 662 keV ^137^Cs gamma rays with inorganic cesium lead bromide SC-based detectors prepared by the melt crystallization method^16^. They further improved the ER to 1.4% with the adoption of asymmetric device design^17^. For solution-processed OIHP SC detectors, although there are still large gaps in terms of stability and ER in gamma-ray detection compared to their inorganic counterparts, there have gained rapid development in recent years. For instance, Yakunin et al. demonstrated the energy spectrum acquisition capability of FAPbI_3_ SCs (ER: 35% for ^241^Am 59.5 keV peak) for the first time in 2016^18^. Wei et al. adopted chloride ions to compensate for the p-type defects in the MAPbBr_3_ perovskite SCs, enabling the improved carrier mobility and bulk resistivity and hence the high ER of 6.5% for ^137^Cs 662 keV peak^19^. In addition, He et al. introduced the asymmetric Schottky junction structure to reduce the dark current in MAPbI_3_ SC radiation detectors, which gives rise to ER of 12% for ^241^Am 59.5 keV and 6.8% for ^57^Co 122 keV γ-ray^20^. This is largely due to the inferior crystalline quality of the SCs grown by the common inverse temperature crystallization (ITC) method^21^. The resultant electronic and ionic defects in the SCs are detrimental to carrier extraction and biasing stability of the detectors, respectively^22–24^. Although higher crystal quality can be achieved by precipitating the crystals in a supersaturated solution under constant temperature^6,11^, it remains challenging to grow wafer-scale SCs with both high uniformity and a fast growth rate through this method.

In this work, we developed a modified space-confined ITC method to grow wafer-scale detector-grade MAPbI_3_ SCs with lateral sizes of up to ~4 cm by introducing a low surface energy perfluorinated-gel (F-gel) modification layer as the soft contact layer on the growth substrates. Near-infrared (NIR) polarized optical microscopy revealed the elimination of the stress during crystal growth as a result of the soft contact between the gel and crystals, which contributes to the significantly improved crystalline quality, as evidenced by the 3.5-fold reduced full-width-at-half-maximum (FWHM) of the (200) plane from the X-ray rocking curve and three times lower trap density. With the adoption of a simple coplanar device structure, the MAPbI_3_ wafer-based detectors display an extremely low dark current of ~1 nA and ultralow baseline drift of 4.0 × 10^−8 ^nA cm^−1^ s^−1^ V^−1^ under a large electric field of up to 1000 V cm^−1^. Ultimately, a record high ER of 4.9% among all solution-processed direct-type radiation detectors was achieved for the 59.5 keV ^241^Am gamma-ray under an ultralow operating bias of 5 V at room temperature, which is also close to that of commercial CZT detectors operated with a much larger working bias.

## Results

### The F-gel-confined growth of the SC wafer

**Fig. 1:**
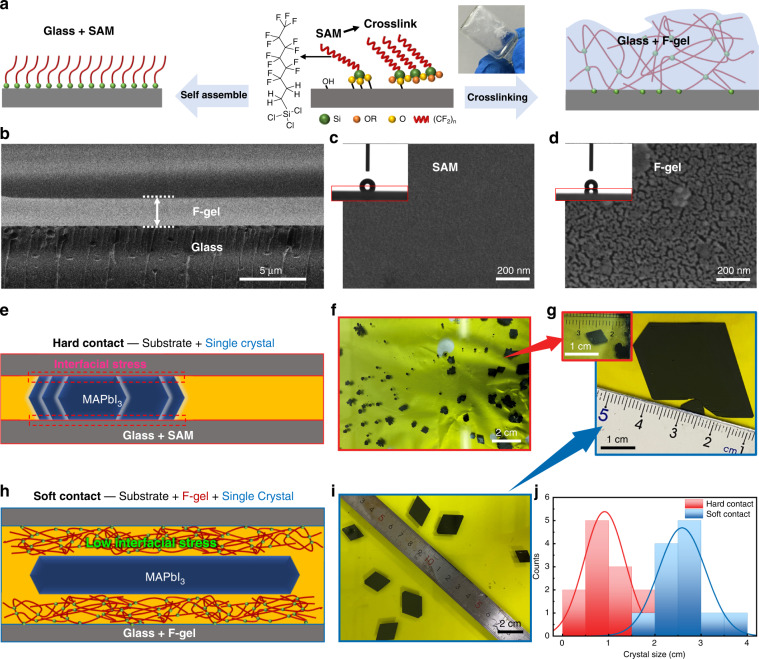
MAPbI_3_ SC growth on different substrates. **a** Schematic diagram of the formation process of F-gel and SAM on the substrate. SEM images of the **b** cross-section of the F-gel, **c** SAM surface and **d** F-gel surface; (inset of (**c**) is the GBL liquid drop on the SAM substrate, inset of (**d**) is the GBL liquid drop on the F-gel substrate). **e** Schematic diagram and **f** picture of crystal growth on SAM substrate. **g** Typical enlarged photos of crystal growth on SAM (top) and F-gel (bottom) substrates. **h** Schematic diagram and **i** picture of crystal growth on the F-gel substrate. **j** Size statistics of crystals grown on hard and soft contact substrates

As schematically shown in Fig. 1a, to achieve the soft contact of the F-gel-modified substrates, the perfluorosilane solution was coated on the surface of the glass substrate and then stored in humid ambient conditions for in situ cross-linking of R-Si-OH bonds to form thick films, in contrast to the self-assembly of perfluorosilane monolayers on the substrate, which has been widely used in conventional space-confined ITC crystal growth processes. The microscopic morphology as well as the thickness of the F-gel layer were characterized by scanning electron microscopy (SEM), as shown in Fig. 1b–d and Supplementary Figs. S1 and S2, which show a micron-scale thick F-gel network layer formed on the substrate surface after modification, in contrast to the thin, smooth surface morphology of the self-assembled monolayer (SAM) on the reference substrate. Contact angle tests using water and γ-butyrolactone (GBL) liquid droplets on substrates with various treatments were also performed to characterize the hydrophobicity of the substrates. As shown in the inset of Fig. 1c, d and Supplementary Fig. S3, the F-gel treated substrates show larger contact angles of 117° and 93° for water and GBL solvent, respectively, compared to 90° and 62° for those on the SAM substrates, which is beneficial for promoting the microcirculation of the precursor solution between gel-modified substrates during crystal growth that is important for wafer-scale single-crystal growth.

We propose that compared to conventional hard contact SAM substrates (Fig. 1e), the flexible and smooth F-gel can eliminate the lattice mismatch between the crystal and substrate and alleviate the physical hindrance from the hard substrates during space-confined crystal growth (Fig. 1h). In addition, the low surface energy of perfluorinated molecular chains can suppress the chemical interaction and eliminate stress between the crystal and the substrate, therefore providing a growth environment close to an open system to facilitate large-size and high-quality crystal growth. As shown in Fig. 1f, i, the nucleation centers of the crystals on the F-gel substrate were substantially reduced compared to those on the SAM substrate, making it easier to grow large-sized single-crystal wafers with lateral sizes up to 4 cm with hundreds of microns thickness (Fig. 1g). We further summarize the size of the crystals grown on different substrates. As displayed in Fig. 1j, the F-gel substrates dramatically increased the average size of the crystal wafers by nearly 2.8 times, nearly 10 times in the area, compared to that of the wafers grown with the SAM substrates, which verifies the effectiveness of this strategy in preparing large-sized perovskite SC wafers.

**Fig. 2:**
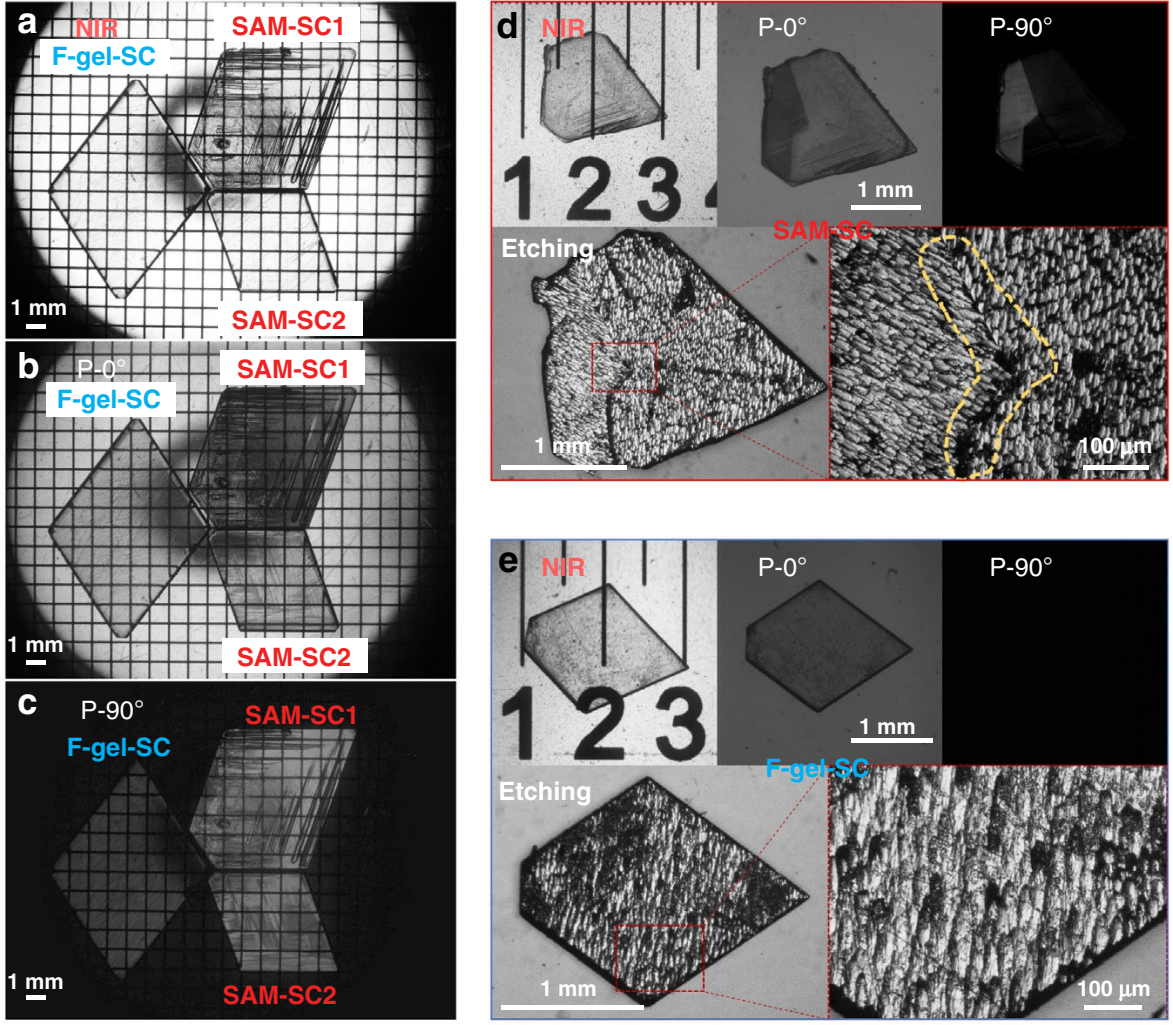
NIR optical microscopy characterization of MAPbI_3_ SCs. NIR optical microscopy images of crystals **a** without polarizers, **b** with parallel aligned polarizers and **c** with vertical aligned polarizers in the transmission mode. (F-gel-SC is grown on a soft contact substrate; SAM-SC1 is grown on a hard contact substrate; SAM-SC2 is grown on a hard contact substrate and its surface is polished.) **d** Polarized NIR images of crystals grown on hard substrates (top) and visible light images of crystal surfaces etched by acetone (bottom). **e** Polarized NIR images of crystals grown on soft substrates (top) and visible light images of crystal surfaces etched by acetone (bottom)

To assess the crystal quality of the as-grown SC wafers, which is especially important for radiation detection performance, we carried out NIR optical microscopy characterization on the crystals, which is able to visualize the macroscopic internal defects in opaque narrow band gap materials such as MAPbI_3_. Figure 2a shows the NIR transmission images of typical crystals grown on the F-gel substrate (F-gel-SC) and on the SAM substrate (SAM-SC). It is observed that the F-gel-grown SCs always show clear crystal transparency with high uniformity under NIR imaging, in strong contrast to the poor transparency and a large amount of macroscopic defects in those grown on SAM substrates (SAM-SC1). These defects are mainly located on the surface of the crystals, since they can be removed after polishing the crystal surface (SAM-SC2), which results in high transparency to NIR light similar to the F-gel-grown crystal. However, there was still a significant difference in the domain distribution observed in the SCs when we further added polarizers in the imaging system with various polarization angles. It is shown in the polarized optical microscope images in Fig. 2b, c that the crystals grown on the hard contact substrate have interlaced domains with different brightnesses inside the crystals (SAM-SC1 and SAM-SC2), which are known as ferroelastic domains with different orientations that are usually generated under large external stress^25^, while the crystals grown on the soft contact interface possess a unidirectional domain distribution without any domain boundaries (as in the F-gel-SC in Fig. 2b, c). To further verify the domain distribution in the crystals observed in the polarized optical microscope, we etched the surface of the crystals grown on different substrates by soaking the crystals in acetone for approximately 2 min to directly expose the domains in the crystal^26^. As shown in Fig. 2d, domains with interlaced orientations can be clearly observed in the crystals grown on SAM substrates after etching, in good accordance with the two domain orientations in the polarized NIR images. For soft contact grown crystals, a periodic ordered domain with a unitary orientation was observed on the surface after etching (Fig. 2e), which also matches well with the polarized NIR imaging results. This result indicates that the external stress from the hard substrate is dramatically relieved by the soft F-gel layer during the space-confined crystal growth process, which not only prevents the mechanical deformations of the SCs and hence suppresses defect formation on the crystal surface but also improves the domain orientation uniformity in the crystal interior over a large scale. As a result, the crystals can be simultaneously prepared with both fast growth speed and high crystal quality on the F-gel substrates, which also enables the direct solution-processed growth of SC wafers instead of cutting and polishing them from large ingots that are always required in wafer fabrication of other crystals, such as CZT or silicon.

**Fig. 3:**
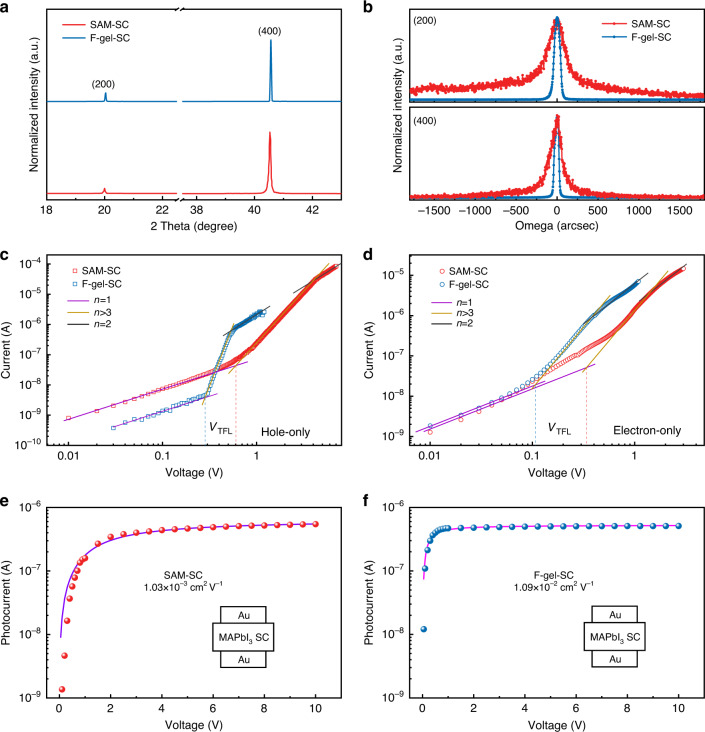
Crystallinity and charge transport properties of MAPbI_3_ SCs. **a** XRD and **b** XRD rocking curves for crystals grown on different substrates. **c** SCLC tests of hole-only and **d** electron-only devices for crystals grown on different substrates. The *μτ* product derived from the steady-state photocurrent method for crystals grown on **e** SAM and **f** F-gel substrates

The crystalline quality of the SCs was further evaluated by X-ray diffraction (XRD) measurements. As shown in Fig. 3a and Supplementary Fig. S4, both kinds of SCs exhibit sharp diffraction peaks with (200) and (400) orientations. The high-resolution X-ray rocking curves of the (200) and (400) peaks of the two kinds of SCs are shown in Fig. 3b, which exhibited very narrow FWHMs of only 84.96 and 74.42 arcsec for the F-gel-SC, respectively, in contrast to 300.06 and 171.72 arcsec for the SAM-SC, further confirming the excellent quality of our monocrystalline wafers offering the potential to achieve excellent radiation detection performance.

The superior crystallinity and uniformity of the F-gel-grown crystals compared to the SAM-grown crystals are expected to dramatically improve their overall charge transport properties, which was assessed by measuring the carrier mobility and trap density change of the SCs with the space-charge-limited-current (SCLC) method (Fig. 3c, d). The device configurations for the SCLC measurements are Cu/bathocuproine (BCP)/C_60_/MAPbI_3_ SC/C_60_/BCP/Cu and Au/MAPbI_3_ SC/Au for electron- and hole-only devices, respectively. The trap density (*n*_t_) can be determined from the formula^27^:

$$V_{{{{\mathrm{TFL}}}}} = \frac{{en_tL^2}}{{{{{\mathrm{2}}}}\varepsilon \varepsilon _0}}$$

where *ε* and *ε*_0_ are the relative dielectric constant of MAPbI_3_ and the vacuum permittivity, respectively; *e* is the elementary charge; and *L* is the thickness of the SC. For F-gel-SC, the electron and hole trap densities were calculated to be 2.18 × 10^10^ and 3.06 × 10^10 ^cm^−3^, respectively, which are 3.3 times lower than those of SAM-SCs (electron trap density: 7.13 × 10^10 ^cm^−3^, hole trap density: 9.44 × 10^10 ^cm^−3^). The defect density of the crystals is comparable to that of silicon SCs grown by Czochralski method with multiple high-temperature melting processes. Meanwhile, the electron and hole mobilities (*μ*) of the crystals were derived from the Mott–Gurney Law^6^:

$$J_{{{\mathrm{D}}}} = \frac{{{{{\mathrm{9}}}}\varepsilon \varepsilon _0\mu V^2}}{{8L^3}}$$

where *J*_D_ is the current density and *V* is the bias voltage. The electron and hole mobilities of the SCs also dramatically increased from 32.07 to 110.90 cm^2^ V^−1^ s^−1^ and from 42.11 to 97.15 cm^2^ V^−1^ s^−1^, respectively, with the adoption of F-gel substrates. Notably, the MAPbI_3_ SCs exhibit balanced electron and hole mobilities, which is beneficial for efficient charge carrier collection during the radiation detection process. We further measured the mobility-lifetime (*μτ*) product of the SCs with the steady-state photocurrent method, which is the key quantity of merit to evaluate the charge collection capabilities of radiation detection materials. The *μτ* product is obtained by fitting the photocurrent-voltage curve (Fig. 3e, f) by the Hecht equation^18,28^:

$${\it{I}}{{{\mathrm{ = }}}}\frac{{I_0\mu \tau {\it{V}}}}{{L^2}}\left( {{{{\mathrm{1}}}} - {{{\mathrm{exp}}}}\left( { - \frac{{L^2}}{{\mu \tau {\it{V}}}}} \right)} \right)$$

where *I*_0_ is the saturated photocurrent and *V* is the applied bias. Benefiting from the increased carrier mobility and suppressed trap density, the F-gel-SC possess a higher *μτ* product of 1.09 × 10^−2^ cm^2^ V^−1^, which is ~10 times larger than those of the SAM-SC and is also one of the highest reported values for MAPbI_3_ SCs grown by the ITC method to the best of our knowledge. All the above results indicate that the F-gel-confined crystal growth method is effective in the fabrication of detector-grade OIHP single-crystal wafers with both high crystalline quality and superior electrical transport properties.

### Radiation detection performance of the SC devices

**Fig. 4:**
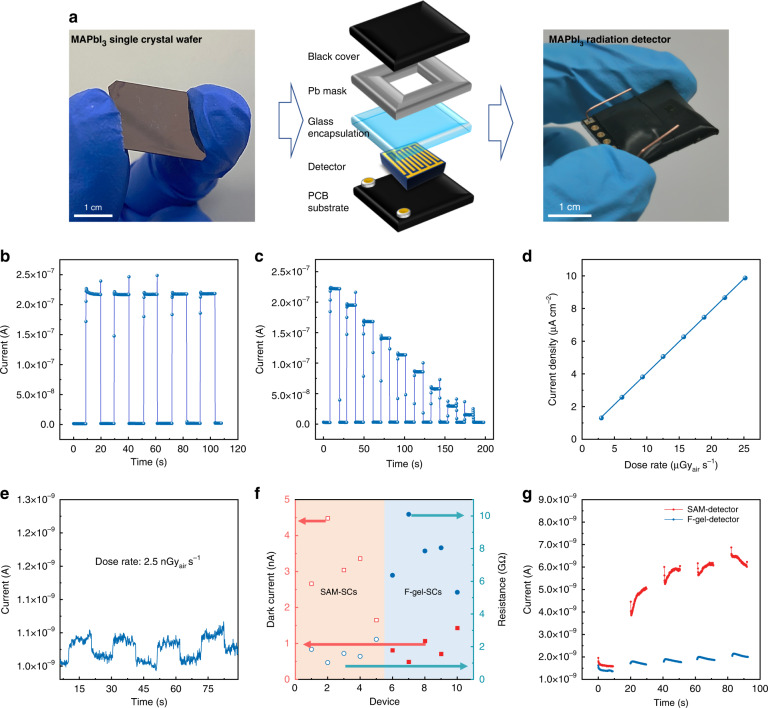
X-ray detection performance of the MAPbI_3_ SC devices. **a** Photographs of F-gel-SC wafer (left), the corresponding radiation detector (right), and the schematic diagram of the device structure (middle). **b** The current response curve of the F-gel-detector when successively turning the X-ray on and off. **c** Current response curve of the F-gel-detector under various X-ray dose rates while successively turning on and off the X-ray. **d** Photocurrent variation as a function of X-ray dose rate of the F-gel-detector from (**c**). **e** Photocurrent response curves of the F-gel-detector measured at an ultralow X-ray dose (2.5 nGy_air_ s^−1^). **f** The statistics of dark current at 5 V bias and the corresponding resistance of crystals grown on different substrates. **g** The dark current variation after multiple light-dark cycles with X-ray irradiation

With the outstanding electrical properties of the F-gel- SCs, we prepared radiation detectors based on them with a metal-semiconductor-metal coplanar device architecture, which has been proven to possess both high X-ray sensitivity and low working bias^29^. The crystal surface was covered with a thin aminosilane layer before Au electrode deposition to inhibit the interfacial-electrochemical reaction, as we reported previously^22^. After electrode deposition, the devices were encapsulated by the cover glass and attached to a printed circuit board (PCB) substrate for bonding and wire connection (Fig. 4a). Finally, the Pb mask was attached to the device to define the effective working area, and a black tape was wrapped on top to shield the device from the influence of environmental light.

We first examined the X-ray response of the devices under an X-ray source with energy up to 50 keV. Figure 4b and Supplementary Fig. S5 show that both the F-gel-SC-based detectors (F-gel-detectors) and the SAM-SC-based detectors (SAM-detectors) achieve an excellent current response of approximately 10 μA cm^−2^ under a dose rate of 25 μGy_air_ s^−1^ at a 5 V bias, indicating that both possess extremely high X-ray responsivity. Furthermore, the X-ray photocurrent density of the F-gel SC devices under a 5 V bias was measured with a gradually decreasing incident dose rate (Fig. 4c), and the device sensitivity was derived from the slope of the dose-rate-dependent photocurrent curve in Fig. 4d. F-gel-detectors exhibited a large average sensitivity of 3.0 × 10^5^ μC ${{\mathrm{Gy}}^{-1}_{\mathrm{air}}}$ cm^−2^, which remarkably outperforms benchmark commercial detectors such as CZT and is also among the highest reported values for perovskite-based radiation detectors.

**Fig. 5:**
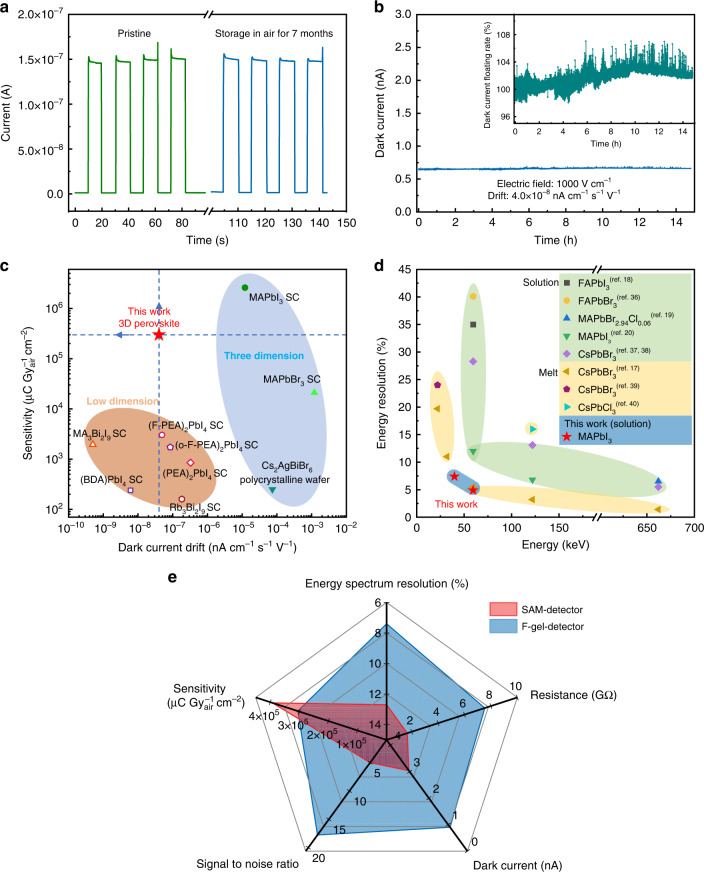
Device stability and performance comparison. **a** Storage stability of F-gel-detector. **b** Dark current output as a function of time for the devices based on F-gel-SC under 1000 V cm^−1^ electric field (inset: dark current floating rate). **c** Summary of the sensitivity and dark current drift of various kinds of MHP radiation detectors. **d** ER of the reported MHP-based radiation detectors. **e** Radiation detection performance comparison between the F-gel-detectors and SAM-detectors

Dark current suppression and stabilization are as important as signal enhancement in gamma-ray spectroscopy. We measured the dark current of the F-gel-detectors with that of the SAM-detectors at a 5 V bias, and the representative *I-V* curves are shown in Supplementary Fig. S6a. As summarized in Fig. 4f, the average dark current of F-gel-detectors is 0.90 nA, in contrast to 3.04 nA for the SAM-detectors. As a result, the average resistance of the F-gel-detectors is as high as 7.54 GΩ, which is nearly 5 times larger than that of the SAM-detectors (1.67 GΩ). Such an obvious difference is mainly caused by the lower trap density in the F-gel-SC that suppressed the background self-doping concentration. The improved crystal quality of the F-gel-SC also suppressed the ionic defect-induced ion migration channels, which led to better biasing stability. As shown in Fig. 5b and Supplementary Fig. S7, the devices based on F-gel-SCs exhibit an ultralow baseline drift of 4.0 × 10^−8 ^nA cm^−1^ s^−1^ V^−1^ under a large electric field of 1000 V cm^−1^, which is among the best biasing stabilities reported for three-dimensional OIHP SCs and even approaches those of zero-dimensional ones, while the sensitivity is more than 2 orders of magnitude larger than them (Fig. 5c and Table 1)^9,23,30–35^. In addition, after successively switching on and off of the X-ray irradiation, the change rate of dark current was 28% for F-gel-detectors after 5 cycles, which is 10 times smaller than the that of the SAM-detector (Fig. 4g). Benefiting from the high sensitivity, low dark current and excellent biasing stability, F-gel-detectors are able to distinguish the weak signal from noise under an ultralow X-ray dose rate of 2.5 nGy_air_ s^−1^ with a signal-to-noise ratio (SNR) of 4.6 (Fig. 4e), which is especially desirable for gamma-ray detection in view of the very low photon flux of approximately 10^4^ counts s^−1^ cm^−2^ for common gamma-ray sources^17^. In addition, the detectors also demonstrate a fast response speed with a rise time of 7.9 μs and fall time of 13.1 μs (Supplementary Fig. S8), which is fast enough to perform event-by-event analysis in pulse detection mode of common gamma-ray spectroscopy. The encapsulated device exhibited excellent storage stability, with the photocurrent response remaining almost constant after 7 months of storage in the atmosphere (Fig. 5a). We have also traced the irradiation stability of the devices, which exhibited even higher photocurrent response after a continuous radiation exposure of about 32.4 mGy_air_ (Supplementary Fig. S6b). Overall, the optimal radiation detectors demonstrate high sensitivity, large resistivity, low noise, excellent biasing stability and fast response speeds that are difficult to achieve simultaneously in solution-processed radiation detectors thus far, suggesting their bright prospects for high-resolution gamma-ray spectroscopy applications.

**Table 1:** Dark current drift for part of the reported perovskite-based X-ray or γ-ray detectors

Materials	Time (h)	Sensitivity (µC ${{\mathrm{Gy}}^{-1}_{\mathrm{air}}}$ cm^−2^)	Dark current drift (nA cm^−1^ s^−1^ V^−1^)	References
3D MAPbI_3_ single crystal	~1	2.6 × 10^6^	1.2 × 10^−5^	^23^
3D BiOBr-Cs_2_AgBiBr_6_ polycrystalline wafer	~0.1	250	7.4 × 10^−5^	^35^
3D MAPbBr_3_ single crystal/Si integration	–	2.1 × 10^4^	1.2 × 10^−3^	^9^
3D MAPbI_3_ single crystal	~17.5	–	2.0 × 10^−3^	^30^
2D (PEA)_2_PbI_4_ single crystal	~23	–	1.9 × 10^−7^	
0D MA_3_Bi_2_I_9_ single crystal	~24	1947	5.0 × 10^−10^	
2D (PEA)_2_PbI_4_ single crystal	~25	848	3.25 × 10^−7^	^31^
2D (o-F-PEA)_2_PbI_4_ single crystal	~25	1724.5	8.48 × 10^−8^	
2D Rb_3_Bi_2_I_9_ single crystal	~0.5	159.7	1.82 × 10^−7^	^32^
2D (BDA)PbI_4_ single crystal	~14	242	6.06 × 10^−9^	^33^
2D (F-PEA)_2_PbI_4_ single crystal	~2	3402	4.9 × 10^−8^	^34^
3D MAPbI_3_ single crystal	~15	3.0 × 10^5^	4.0 × 10^−8^	This work

### Gamma-ray spectroscopy

**Fig. 6:**
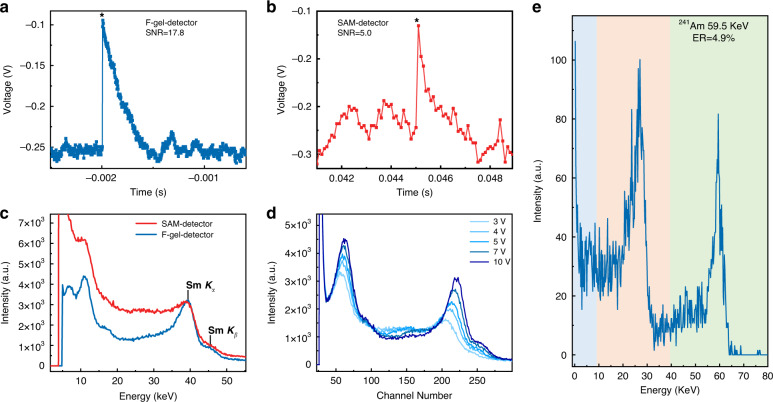
Gamma-ray detection performance of the MAPbI_3_ SC devices. The SNRs of **a** the F-gel-detector and **b** SAM-detector under ^241^Am gamma rays. **c** The energy spectrum measurement with the SAM-detector and F-gel-detector under a ^152^Eu gamma-ray source at room temperature. **d** The energy spectrum of the F-gel-detector with different bias voltages under a ^152^Eu gamma-ray source. **e** The energy spectrum of the ^241^Am source measured with the optimal F-gel-detector

In contrast to the charge integration mode used for X-ray detection, the pulse operation mode is generally required for gamma-ray spectrum acquisition to differentiate a single photon event under a very low incident flux and hence to obtain the intensity versus energy statistics of the radiation source. As a result, a large SNR is crucial for efficient and high ER gamma-ray spectroscopy. The ^241^Am gamma-ray source was used to evaluate the SNR of the devices in gamma-ray spectroscopy. The output signals from the preamplifier of the F-gel-detectors and SAM-detectors are recorded under gamma-ray irradiation, which shows a larger SNR of 17.8 for the F-gel-detectors, in contrast to the small SNR of 5.0 for the SAM-detectors (Fig. 6a, b). As a result, the noise-induced peak broadening and tailing of the energy spectrum can be greatly suppressed with F-gel-detectors, which leads to better ER.

To confirm this, the energy spectrum measurement is carried out utilizing a ^152^Eu gamma-ray source at room temperature and atmospheric conditions with a bias of 5 V and a forming time of 200 s. As shown in Fig. 6c, the signal peaks in the energy spectrum are sharp and distinct. Even *K*_*α*_ and *K*_*β*_ peaks of Sm are clearly visible between 30 and 50 keV for the F-gel-detectors, and the ER is calculated to be 7.4% for the characteristic peak of 39.8 keV. For the SAM-detectors, the 39.8 keV peak is merged into the noise-induced low-energy tailing that can hardly be distinguished, which leads to a poor ER of 12.7%. We also evaluated the influence of the working bias of the devices on the measured energy spectrum. As shown in Fig. 6d, a substantially higher peak intensity of 39.8 keV and better ER are achieved when increasing the bias voltage due to the more efficient collection of photon-generated charge carriers. The increase in the channel number of the peak tends to saturate with increasing bias up to 10 V, suggesting that full charge collection can be achieved even under such a small bias in our coplanar structured detectors, in contrast to sandwich structured commercial CZT detectors, which usually require hundreds to thousands of volts to work. The ultralow working bias of the detectors not only reduces energy consumption and expenses but also enhances the portability and safety of the detection system.

Finally, we measured the ^241^Am source energy spectrum with the optimal F-gel-detector. As shown in Fig. 6e, a record resolution of 4.9% for the 59.5 keV peak was achieved under 5 V bias, which is the highest value for MHP-based gamma detectors at the same energy range as well as for solution-processed semiconductor gamma-ray detectors ever reported (Fig. 5d)^17–20,36–40^, and even approaches that of commercial CZT detectors. The excellent ER is mainly attributed to the lower dark current, larger resistance, and better SNR of the F-gel-detectors compared to the SAM-detectors (Fig. 5e), which further verified the effectiveness of F-gel in the preparation of detector-grade high-quality OIHP SCs.

## Discussion

We designed and prepared soft contact substrates based on a cross-linked F-gel network for the growth of OIHP SCs and obtained large-size detector-grade MAPbI_3_ SC wafers using the ITC space-confined solution growth method. The soft contact substrate can relieve the external stress on the crystals and eliminate the interaction between the crystal and the glass substrate during the crystal growth process, thus allowing the crystal to grow in an environment close to open space. The resultant SCs show a larger lateral size, lower surface defect density, and unitary internal domain orientation, as revealed by polarized NIR optical microscopy. In addition, the FWHM of the (200) plane of the F-gel-SCs from the X-ray rocking curve was reduced by 3.5 times compared to that of the hard contact-grown ones. The excellent crystal quality leads to superior electric transport properties with a high *μτ* product reaching 1.09 × 10^−2^ cm^2^ V^−1^, which is ~10 times larger than the control values. Ultimately, the coplanar structured gamma-ray detectors were constructed and exhibited a low and stable dark current output below 1 nA with a baseline drift down to 4.0 × 10^−8 ^nA cm^−1^ s^−1^ V^−1^ under a large electric field of 1000 V cm^−1^. A well-defined ^241^Am gamma-ray spectrum was collected with a record high ER of 4.9% for a 59.5 keV peak at only 5 V bias, which benefited from the low dark current, high charge collection efficiency, large SNR, and excellent biasing stability realized simultaneously in the optimal detectors. The high-resolution gamma-ray spectroscopy under ultralow working voltage with the low-cost solution-processed radiation detectors demonstrated here will be of great significance for industrial and biomedical radiation detection applications in the future. Moreover, the gel-confined solution growth method presented here provides a promising strategy to prepare large-sized MHP SC wafers directly without conventional cutting and polishing procedures, which is also desirable for other MHP-based optoelectronic devices, such as solar cells, light emitting diodes and photodetectors. Further work should be focused on the further suppression of defect density and improvement of the uniformity of the large-sized single-crystal wafers through the composition, dopant and strain engineering, in order to eventually meet the stringent requirements for photon counting applications.

## Materials and methods

### Preparation of MAPbI_3_ single crystals

High-quality MAPbI_3_ SCs were grown using the modified inverse temperature space-confined growth method. For the preparation of the SAM-based substrate, the flat glass is washed and placed in a 1% perfluorinated silane IPA solution, soaked for 30 min and then rinsed with a large clean IPA to remove excessive unreacted perfluorinated silane in a dry environment. For the fabrication of the F-gel-based substrate, the silane solution is coated in the air (40–50% humidity) on a flat and clean glass surface and left in the air for 5 min. The above procedure is repeated 3–5 times to obtain a cross-linked network of F-gel structures covering the glass surface. The crystal growth process is similar to that described in our previous report^22,41^. For the preparation of the perovskite precursor solution, equal molar amounts of MAI and PbI_2_ were dissolved in GBL solvent at a concentration of 1.5 M and stirred for more than 4 h at approximately 70 °C. The precursor solution was added between two pieces of glass and placed on a 70 °C hot stage for 2 h; then, the temperature was increased to 120 °C for crystal growth. Finally, the crystals were removed from the substrates and stored in a nitrogen atmosphere.

### Device preparation

Crystals with similar sizes were selected and fixed on a glass substrate, followed by spin coating of 3-aminopropyl triethoxysilane on the surface of the crystals as a barrier layer to eliminate interfacial-electrochemical reactions. An interdigital electrode was prepared with a finger width of approximately 100 μm and a finger space of approximately 50 μm. The device was then encapsulated by a cover glass and a lead mask covered on top with a 1.5 × 1.5 mm^2^ effective area, and this effective area is used for all detector performance demonstrations in this article. Finally, the device was shaded and integrated onto a PCB board for wire bonding and operation.

### Material characterization

The microscopic surface morphology and cross-sectional morphology of the substrates with different treatments were collected with field emission SEM (HITACHI, SU8020). The polarized NIR imaging system was used to visualize the macroscopic defects and internal domain distribution in the SCs, which consists of a 980 nm infrared light source coupled with an optical microscope, a CCD camera, and infrared polarizers. The contact angle data for different substrates were obtained by Dataphysics OCA20 equipment. The SCLC measurement of the SCs was performed by a Keithley 2400 source meter in an N_2_-filled glove box. For F-gel-SCs, the trap-filled limit voltage (*V*_TFL_) is 0.104 and 0.28 V in electron- and hole-only devices with crystals thicknesses of 130 and 180 µm, respectively; and for SAM-SCs, the *V*_TFL_ is 0.34 and 0.60 V in electron- and hole-only devices with crystals thicknesses of 130 and 150 µm, respectively. The μτ measurement of the 620-μm thick F-gel-SC and 610-μm thick SAM-SC was performed by a Keithley 2400 source meter with a 365 nm UV Led with a power density of ~0.6 mW cm^−2^ in the N_2_-filled glove box. The XRD spectra were obtained using an X-ray diffractometer (Empyrean) equipped with a Cu tube operated at 40 kV and 30 mA. The single-crystal rocking curves were tested by a Rigaku Ultima IV X-ray diffraction system with Cu Kα radiation (λ = 0.154056 nm).

### Radiation detector characterization

For the X-ray detection measurement, the X-ray was generated from an Amptek Mini-X2 tube with an Ag target with energy up to 50 keV. The X-ray dose rate was gradually decreased by adjusting the tube current from 80 to 5 μA as well as by inserting metal attenuators with various thicknesses and calibrated by a Radcal Accu-Gold^+^ 10 × 6–180 ion chamber dosimeter. The current response of the devices was recorded by a Keithly 2400 digital source meter at 5 V bias. The SNR of the detectors was tested with a Keysight DSOX3104A oscilloscope coupled with a charge-sensitive preamplifier under a ^241^Am gamma-ray source. The response speed of the devices was measured by the transient photocurrent method with a 570 nm LED as the light source modulated by the wave function generator at 1000–10,000 Hz. The gamma-ray energy spectrum was measured at room temperature in atmospheric conditions under a 5 V bias with a forming time of 200 s. The pulse signal originating from ^152^Eu and ^241^Am source irradiation decayed and entered an electronics system, including an Imdetek MA-01A charge-sensitive preamplifier and an Imdetek PSA-01 portable spectrum analyzer, and was finally collected and transformed into an energy spectrum by a computer.

## Supplementary information

Supplementary Information

## Data Availability

The data that support the findings of this study are available from the corresponding author upon reasonable request.
