# Supplementary material for: Detector-grade perovskite single-crystal wafers via stress-free gel-confined solution growth targeting high-resolution ionizing radiation detection
Source: Light Sci Appl. 2023 Apr 3;12:85. doi: 10.1038/s41377-023-01129-y (PMC10068605; doi:10.1038/s41377-023-01129-y)
Supplement: Supplementary file 1 — Supplementary Information [file 41377_2023_1129_MOESM1_ESM.docx]

**Supplementary Information for**

**Detector-Grade Perovskite Single Crystal Wafers via Stress-Free Gel-Confined Solution Growth Targeting High-Resolution Ionizing Radiation Detection**

*Yilong Song^1#^, Lixiang Wang^2#^, Yongqiang Shi^3,4#^, Weihui Bi^1^, Jianwu Chen^3,4^, Mingwei Hao^1^, Anran Wang^1^, Xueying Yang^1^, Yuan Sun^1^, Fan Yu^1^, Liansheng Li^3,4*^, Yanjun Fang^2,5*^, Deren Yang^2^ and Qingfeng Dong^1*^*

^1^State Key Laboratory of Supramolecular Structure and Materials, College of Chemistry, Jilin University, Changchun, 130012, China.

^2^State Key Laboratory of Silicon Materials and School of Materials Science and Engineering, Zhejiang University, Hangzhou, 310027, China.

^3^Beijing Institute of Control Engineering, Beijing, 100190, China.

^4^Science and Technology on Space Intelligent Control Laboratory, Beijing, 100190, China.

^5^Shanxi-Zheda Institute of Advanced Materials and Chemical Engineering, Taiyuan, 030024, China.

^*^E-mail: [*qfdong@jlu.edu.cn*](mailto:qfdong@jlu.edu.cn)*;* [*jkfang@zju.edu.cn*](mailto:jkfang@zju.edu.cn)*; liliansheng502@163.com*

*^#^Y. S., L.W., Y. S.* contribute equally to this work

**
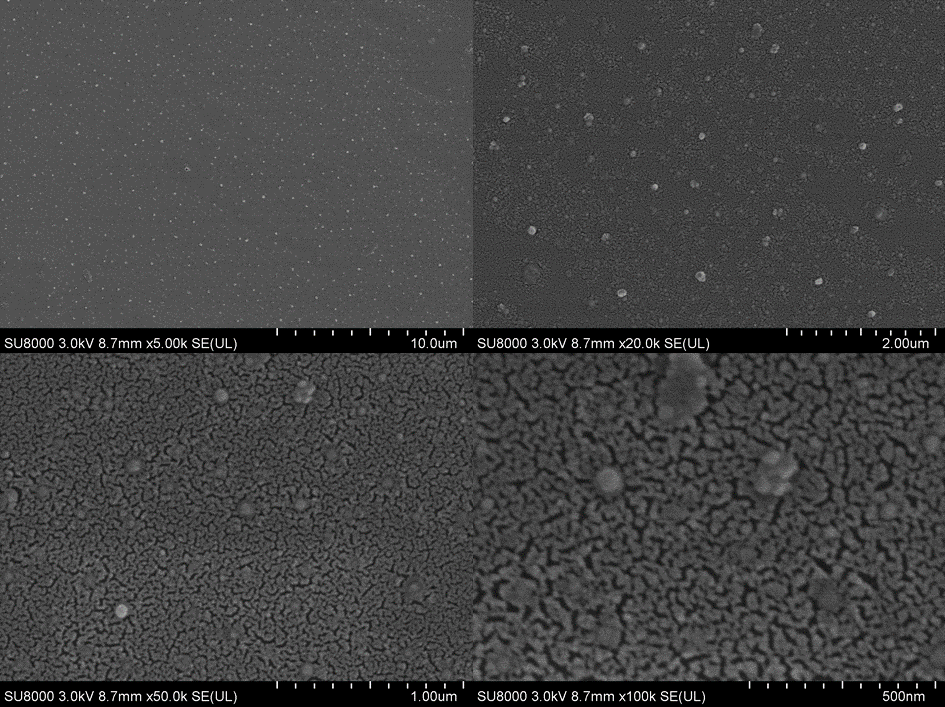
**

**Figure S1.** SEM images of F-gel treated substrates at different magnifications.

**
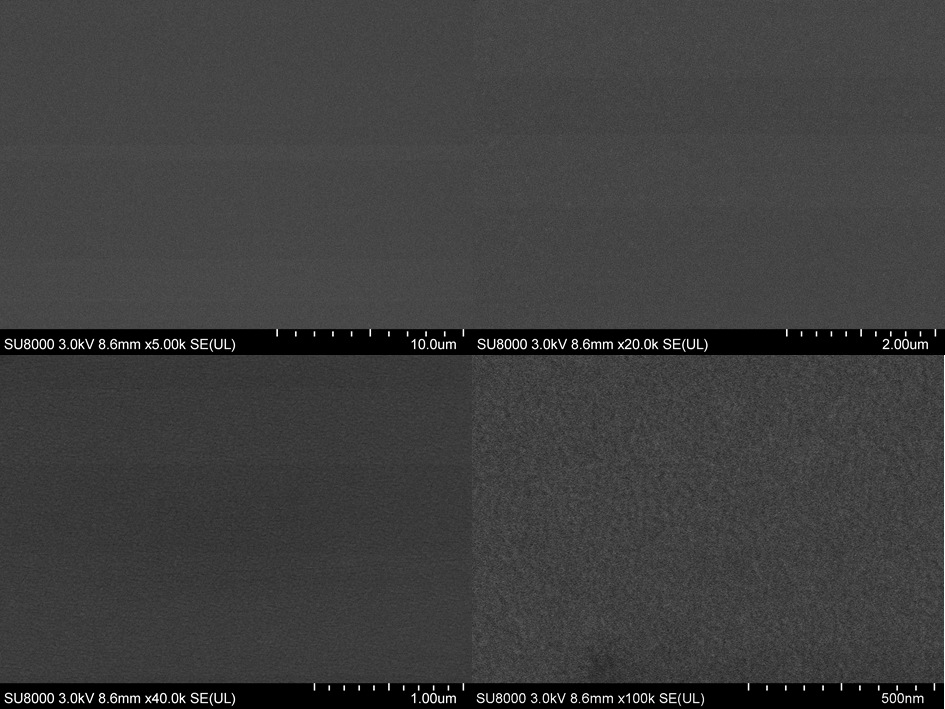
**

**Figure S2.** SEM images of SAM treated substrates at different magnifications.

**
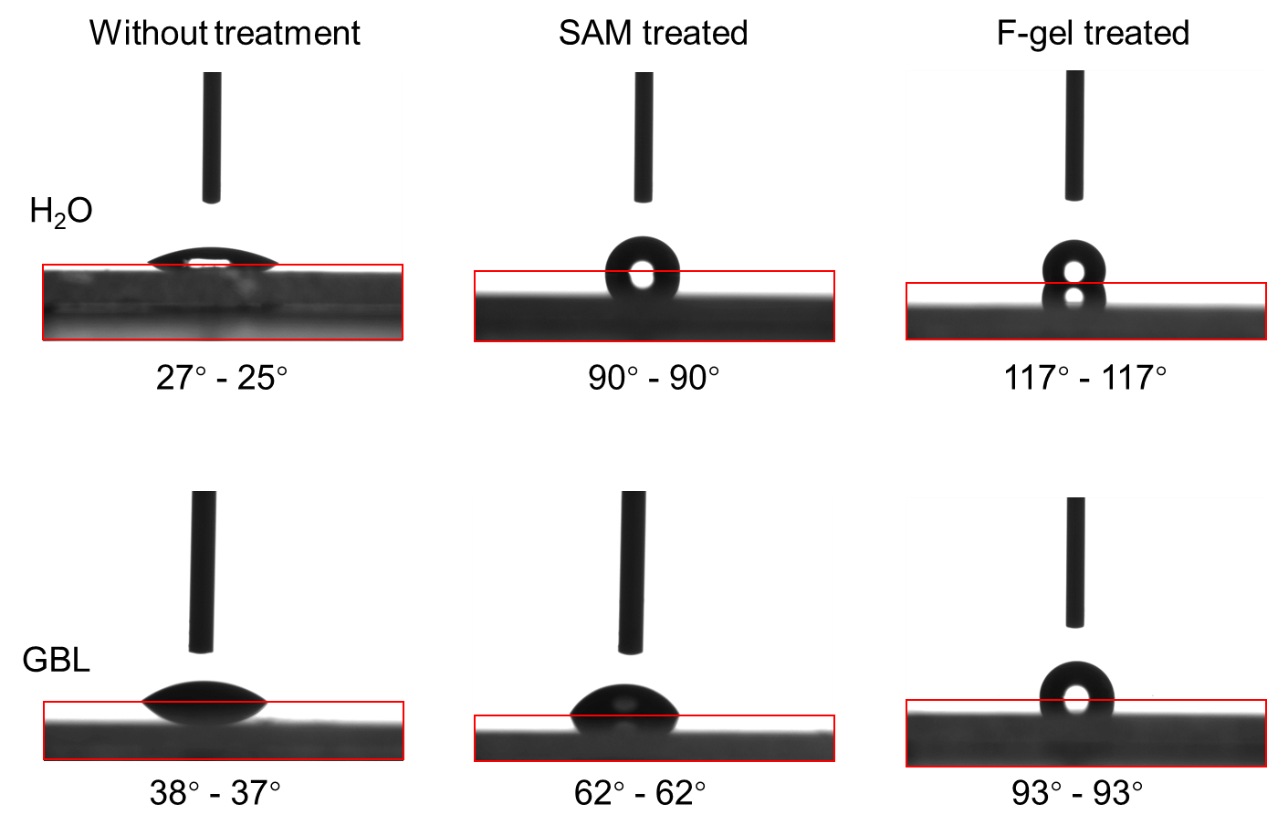
**

**Figure S3.** Contact angle measurement of water and GBL on substrates with different treatments.


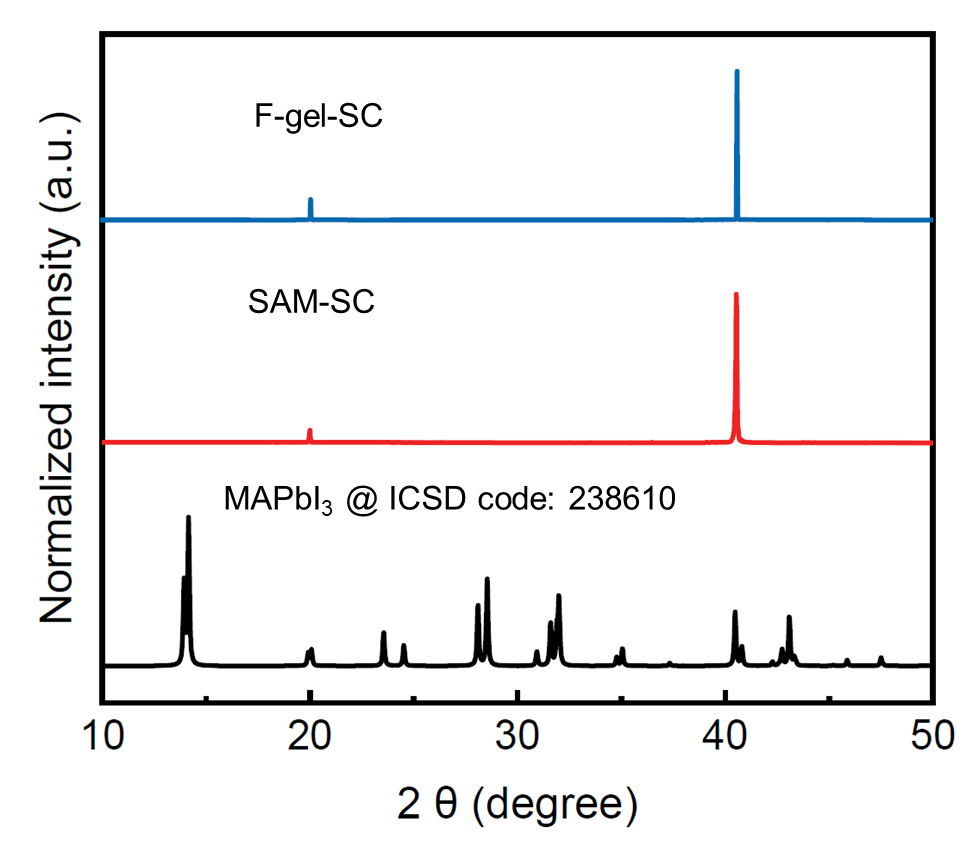


**Figure S4.** Comparison of single crystal XRD spectra with standard MAPbI_3_ XRD spectra (indexed based on ICSD file No. 238610).


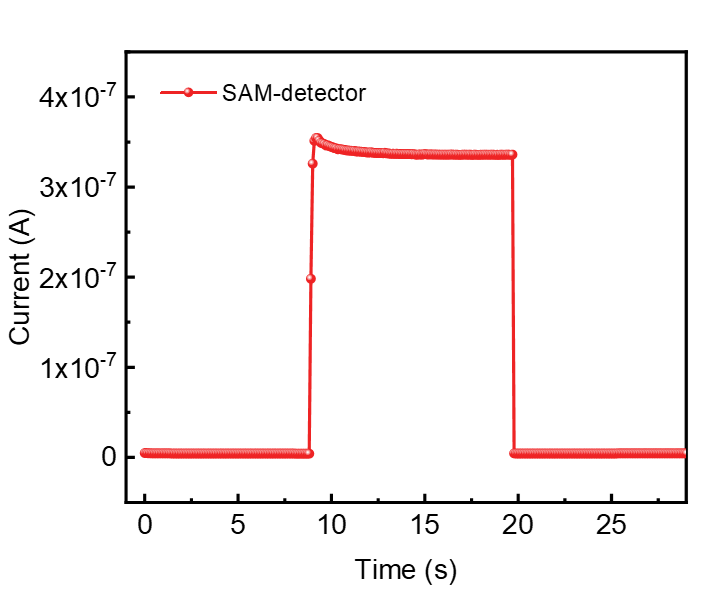


**Figure S5.** X-ray response curves of SAM-detector under the X-ray.

**
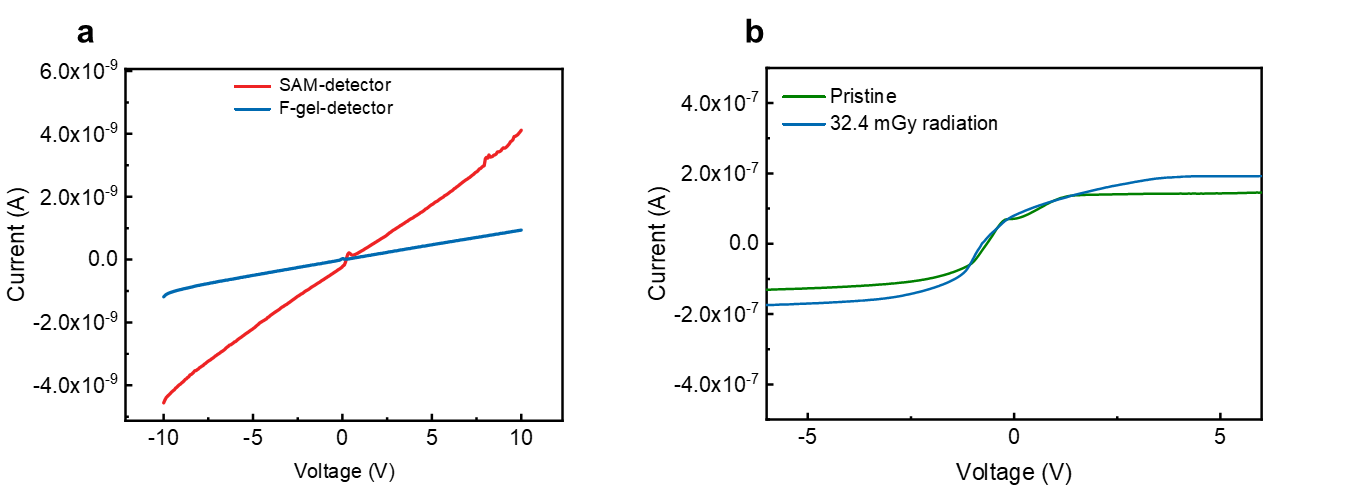
**

**Figure S6.** (a). Dark current curves of F-gel-detector and SAM-detector; (b). Photocurrent comparison of the F-gel-detector before / after X-ray radiation.


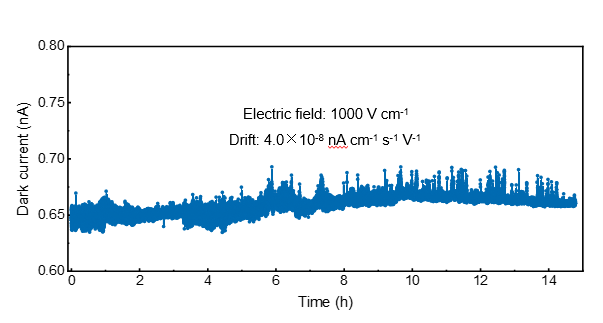


**Figure S7.** Dark current output as a function of time for the devices based on F-gel-SC under 1000 V cm^-1^ electric field.

**
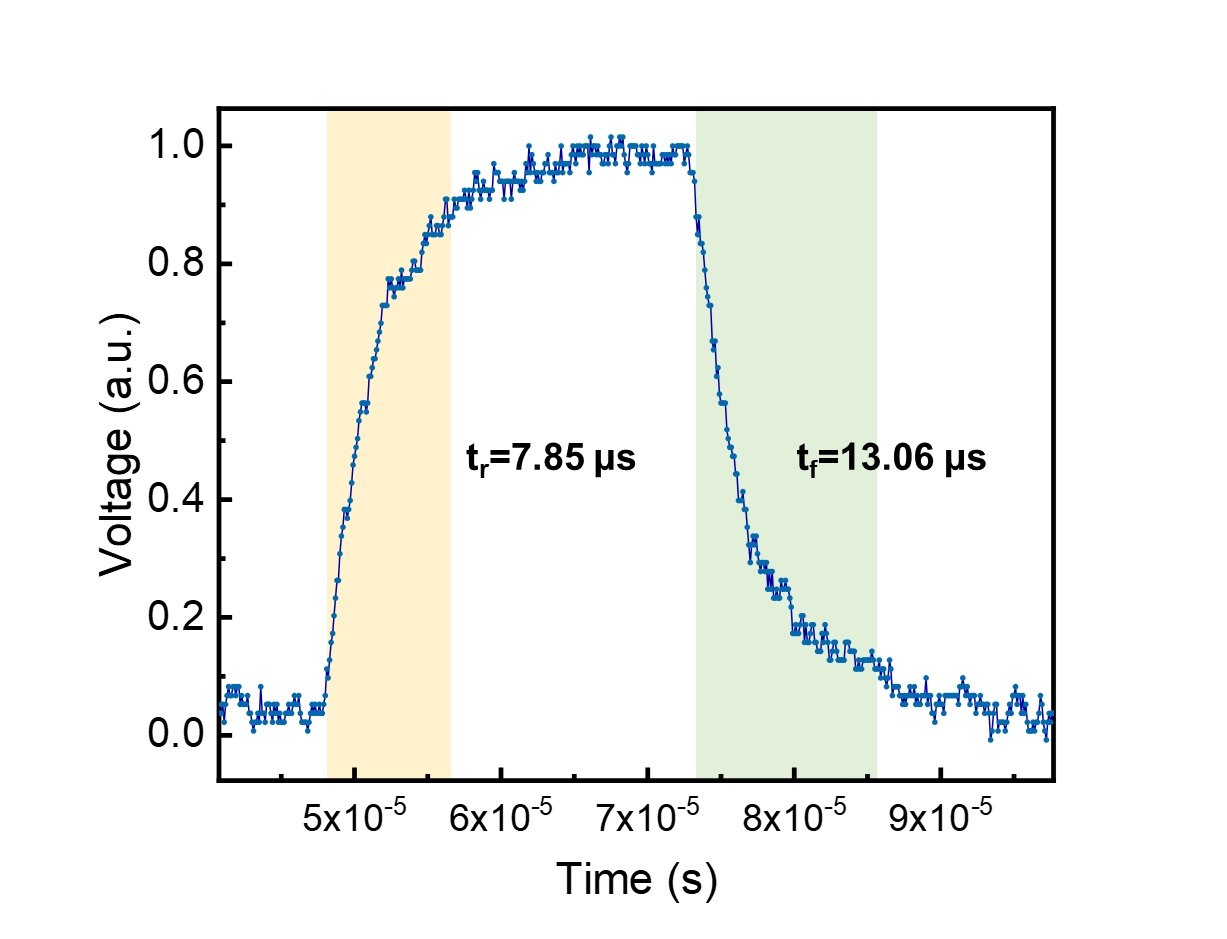
**

**Figure S8.** The response speed curve of F-gel-detector.
